# Therapeutic benefits of proning to improve pulmonary gas exchange in severe respiratory failure: focus on fundamentals of physiology

**DOI:** 10.1113/EP089405

**Published:** 2021-08-13

**Authors:** Ronan M. G. Berg, Jacob Peter Hartmann, Ulrik Winning Iepsen, Regitse Højgaard Christensen, Andreas Ronit, Anne Sofie Andreasen, Damian M. Bailey, Jann Mortensen, Pope L. Moseley, Ronni R. Plovsing

**Affiliations:** ^1^ Department of Biomedical Sciences Faculty of Health and Medical Sciences University of Copenhagen Copenhagen Denmark; ^2^ Department of Clinical Physiology, Nuclear Medicine & PET Copenhagen University Hospital ‐ Rigshospitalet Copenhagen Denmark; ^3^ Centre for Physical Activity Research Copenhagen University Hospital ‐ Rigshospitalet Copenhagen Denmark; ^4^ Neurovascular Research Laboratory Faculty of Life Sciences and Education University of South Wales Pontypridd UK; ^5^ Department of Emergency Medicine North Zealand Hospital Hillerød Denmark; ^6^ Department of Anaesthesia and Intensive Care Copenhagen University Hospital ‐ Hvidovre Hospital Hvidovre Denmark; ^7^ Department of Infectious Diseases Copenhagen University Hospital ‐ Hvidovre Hospital Hvidovre Denmark; ^8^ Department of Anaesthesia and Intensive Care Copenhagen University Hospital ‐ Herlev Hospital Herlev Denmark; ^9^ Department of Clinical Medicine Faculty of Health and Medical Sciences University of Copenhagen Copenhagen Denmark; ^10^ Novo Nordisk Foundation Centre for Protein Research Faculty of Health and Medical Sciences University of Copenhagen Copenhagen Denmark

**Keywords:** acute respiratory distress syndrome, COVID‐19, gas exchange, gravity, respiratory failure, SARS‐CoV‐2

## Abstract

**New Findings:**

- **What is the topic of this review?**
  The use of proning for improving pulmonary gas exchange in critically ill patients.
- **What advances does it highlight?**
  Proning places the lung in its ‘natural’ posture, and thus optimises the ventilation‐perfusion distribution, which enables lung protective ventilation and the alleviation of potentially life‐threatening hypoxaemia in COVID‐19 and other types of critical illness with respiratory failure.

**Abstract:**

The survival benefit of proning patients with acute respiratory distress syndrome (ARDS) is well established and has recently been found to improve pulmonary gas exchange in patients with COVID‐19‐associated ARDS (CARDS). This review outlines the physiological implications of transitioning from supine to prone on alveolar ventilation‐perfusion (${\dot{V}}_{A}\text{--}\dot{Q}$) relationships during spontaneous breathing and during general anaesthesia in the healthy state, as well as during invasive mechanical ventilation in patients with ARDS and CARDS. Spontaneously breathing, awake healthy individuals maintain a small vertical (ventral‐to‐dorsal) ${\dot{V}}_{A}/\dot{Q}$ ratio gradient in the supine position, which is largely neutralised in the prone position, mainly through redistribution of perfusion. In anaesthetised and mechanically ventilated healthy individuals, a vertical ${\dot{V}}_{A}/\dot{Q}$ ratio gradient is present in both postures, but with better ${\dot{V}}_{A}\text{--}\dot{Q}$ matching in the prone position. In ARDS and CARDS, the vertical ${\dot{V}}_{A}/\dot{Q}$ ratio gradient in the supine position becomes larger, with intrapulmonary shunting in gravitationally dependent lung regions due to compression atelectasis of the dorsal lung. This is counteracted by proning, mainly through a more homogeneous distribution of ventilation combined with a largely unaffected high perfusion dorsally, and a consequent substantial improvement in arterial oxygenation. The data regarding proning as a therapy in patients with CARDS is still limited and whether the associated improvement in arterial oxygenation translates to a survival benefit remains unknown. Proning is nonetheless an attractive and lung protective manoeuvre with the potential benefit of improving life‐threatening hypoxaemia in patients with ARDS and CARDS.

## 1 INTRODUCTION

Severe coronavirus disease 2019 (COVID‐19) pneumonia commonly manifests as acute respiratory distress syndrome (ARDS), i.e. acute onset of hypoxaemia and bilateral opacities on chest imaging (Gattinoni et al., 2020; Rello et al., 2020). In the initial reports from China, patients with COVID‐19‐associated ARDS (CARDS) exhibited alarmingly high short‐term mortality rates of 60–70% (Wu et al., 2020; Yang et al., 2020), much higher than those previously reported for severe non‐COVID‐19 ARDS (Bellani et al., 2016). To improve outcome in CARDS, clinical guideline committees were rapidly established and recommendations were based on evidence established in non‐COVID‐19 ARDS (henceforth designated ARDS for simplicity) (Alhazzani et al., 2020; Matthay et al., 2020). These recommendations included lung protective ventilation and placing patients with moderate to severe ARDS in the prone posture for 12–16 h per day. From the early stages of the COVID‐19 pandemic, proning has thus been used for CARDS all over the world (Langer et al., 2021).

Prior to the COVID‐19 pandemic, the impact of proning on intensive care unit (ICU) mortality was investigated in five randomised controlled trials (Gattinoni et al., 2001; Guérin et al., 2004, 2013; Mancebo et al., 2006; Taccone et al., 2009). Of these, the PROSEVA (Proning Severe ARDS patients) trial showed a survival benefit when proning patients with moderate to severe ARDS (Guérin et al., 2013), which was confirmed in a corresponding meta‐analysis with an approximately 25% reduction in 28‐day mortality (Li et al., 2018; Munshi et al., 2017).

However, despite its potential benefits in treating patients with CARDS, the underlying physiological mechanisms remain obscure. Herein, this review outlines the fundamental pulmonary adaptations, focusing primarily on alveolar ventilation‐perfusion (${\dot{V}}_{A}\text{--}\dot{Q}$) relationships, when transitioning (C)ARDS patients from supine to prone.

## 2 PULMONARY MECHANICS IN THE HEALTHY LUNG: SHAPE VERSUS GRAVITY

### 2.1 Upright lung

**FIGURE 1:**
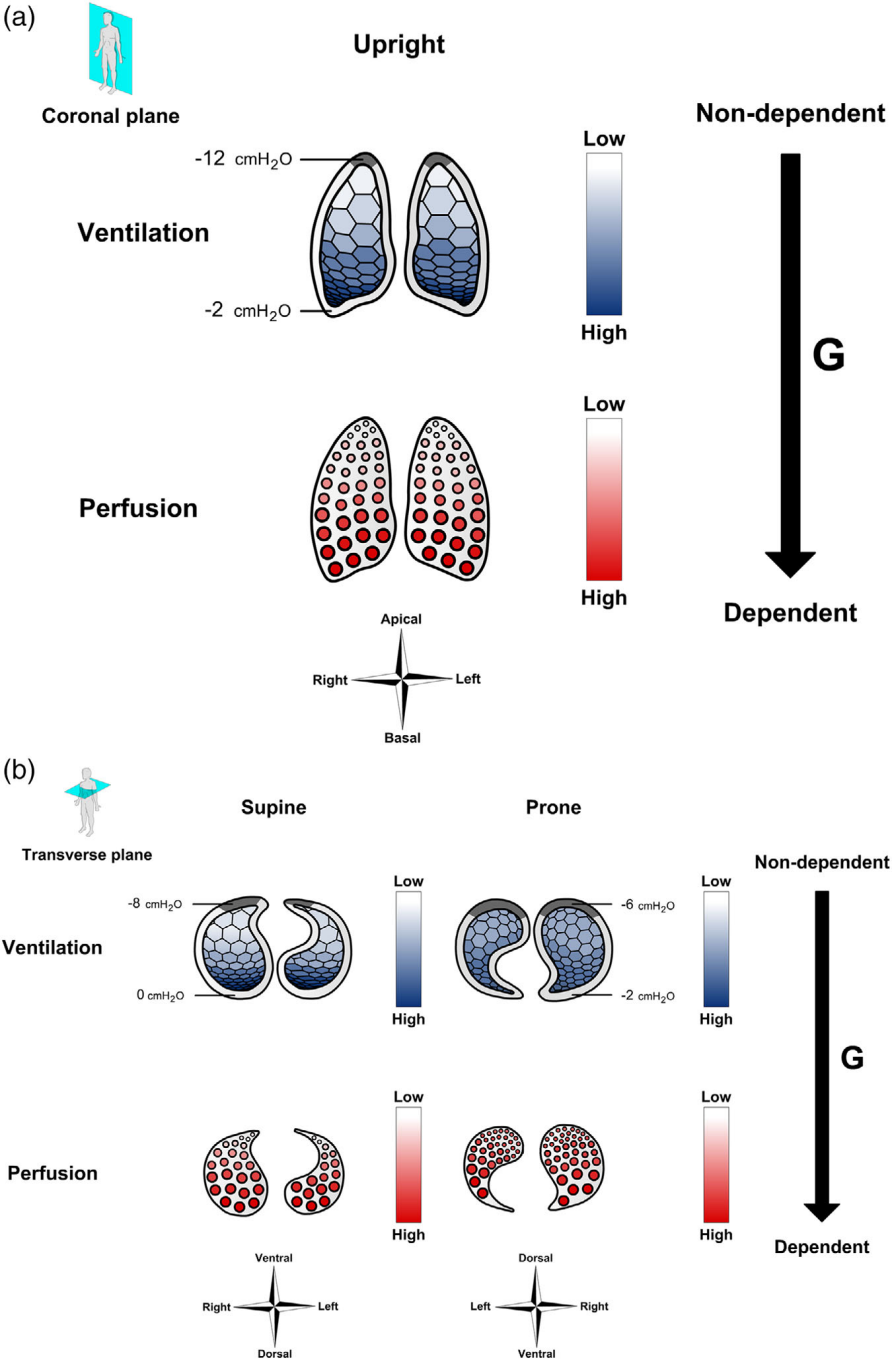
Effects of posture on ventilation and perfusion. (a) The upright lung. A vertical pleural pressure gradient is present, which causes apical alveoli to be more expanded than basal alveoli, and thus ventilation to increase in the apical‐to‐basal direction. Meanwhile, gravity also causes perfusion to increase in the apical‐to‐basal direction. (b) The horizontal lung. In the supine position, both ventilation and perfusion increase in the ventral‐to‐dorsal direction. In the prone position, the change in ventilation is less pronounced because the pleural pressure gradient is halved. In terms of perfusion in the prone position, the higher vascular density in the now non‐dependent dorsal lung regions alleviates the effect of gravity on the perfusion distribution. Pleural pressures are provided for each posture. Grey areas within the pleural space illustrate areas where the lung is ‘constrained’ at the thoracic wall

The upright human lung is shaped as a triangular‐based pyramid, which is conical in the transverse plane with the tip pointing ventrally (Figure 1a). In the excised lung, the alveoli are homogeneously expanded, but because the lungs are suspended in the thoracic cage, which is shaped as an irregular and somewhat rectangular cylinder, alveolar expansion gradually decreases in the apical‐to‐basal direction at functional residual capacity (FRC) in vivo (Vawter et al., 1975). The degree of alveolar expansion is closely related to the pressure in the surrounding pleural space, which is sub‐atmospheric at FRC due to the opposing elastic recoil forces of the lung tissue and the chest wall. This is more pronounced at the apex than at the base of the lung, so that a *vertical* – i.e. parallel to the gravitational vector – pleural pressure gradient of approximately 0.45 cmH_2_O/cm is present (D'Angelo et al., 1970) with a concomitant gradual apical‐to‐basal reduction in transpulmonary pressure (the alveolar‐to‐pleural pressure difference) (Figure 1a).

### 2.2 Horizontal lung

When body position is shifted from upright to horizontal, the direction of the gravitational vector relative to the lungs and chest cage also changes (Figure 1b). Thus, in the supine position, the thorax becomes slightly compressed in the ventral‐to‐dorsal direction, and the abdominal contents are moved cranially, making the thorax ∼20% shorter in the apical‐to‐basal direction (Glazier et al., 1967), so that FRC is reduced by at least 25% (Kaneko et al., 1966; Moreno & Lyons, 1961). A vertical pleural pressure gradient of ∼0.45 cmH_2_O/cm remains with a gradually reduced alveolar expansion in the ventral‐to‐dorsal direction (D'Angelo et al., 1970; Henderson et al., 2013; Tawhai et al., 2009; Wiener‐Kronish et al., 1985) (Figure 1b).

In the prone posture, the direction of the gravitational vector becomes inverted compared to the supine posture (Figure 1b), and FRC becomes ∼10 percentage points higher than in the supine position, thus reaching ∼85% of the upright value (Moreno & Lyons, 1961; Rohdin et al., 2003b), conceivably due to reduced compression of the dorso‐caudal lung regions by the heart and abdominal contents described below. The vertical now dorsal‐to‐ventral pleural pressure gradient is only ∼50% of that observed in both the upright and supine posture (D'Angelo et al., 1970; Henderson et al., 2013; Tawhai et al., 2009; Wiener‐Kronish et al., 1985). Consequently, transpulmonary pressure decreases less in the dorsal‐to‐ventral direction, so that alveolar expansion becomes more homogeneous than in the supine posture (Figure 1b). This is also reflected by posture‐dependent differences in tissue density, i.e., the mass of lung tissue and blood relative to air. Hence, when quantified by either computed tomography (CT) or magnetic resonance imaging (MRI), the prone vertical tissue density gradient is equivalent to 50% of that observed in the supine position (Albert & Hubmayr, 2000; Hoffman, 1985; Kizhakke Puliyakote et al., 2022; Prisk et al., 2007).

### 2.3 Why is the pleural pressure gradient posture‐dependent?

**FIGURE 2:**
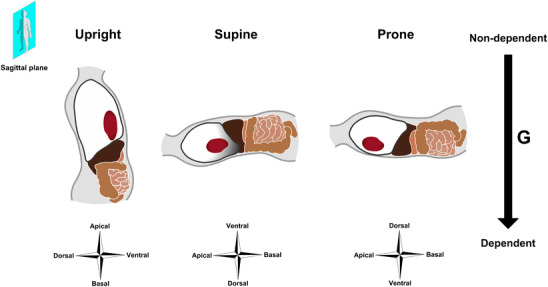
Posture‐dependent displacements of the heart, abdominal contents and diaphragm

While the superimposed hydrostatic pressure on the pleural space exerted by the weight of the lung itself is important for the pleural pressure gradient (West & Matthews, 1972), another factor is the additional hydrostatic pressure imposed by the weight of the heart and abdominal contents (Albert & Hubmayr, 2000) (Figure 2). In the supine position, more than 50% of the lung tissue is below the level of the heart and is to some extent compressed, whereas the heart is resting firmly on the sternum in the prone position (Albert & Hubmayr, 2000). The abdominal contents also contribute by generating an intra‐abdominal hydrostatic pressure, which greatly exceeds alveolar pressure. Since the diaphragm is oriented obliquely in the sagittal plane, this pressure compresses the dorso‐caudal lung regions in the supine, but not in the prone, position (Albert & Hubmayr, 2000). Nevertheless, computer simulations predict that the effect of prone posture on the vertical pleural pressure gradient is present even when these factors are excluded, and appears to depend more on the shape of the lung and thoracic cage relative to the direction of the gravitational vector (Tawhai et al., 2009). Because the lung is triangular in the transverse plane and constrained to remain in contact with the thoracic wall, a greater volume of the lung tissue is ‘fixated’ at the posterior thoracic wall in the prone than at the anterior thoracic wall in the supine position (Figure 1a, b). The gravitational displacement of the lung tissue and thus the vertical pleural pressure gradient is consequently smallest in the prone lung (Tawhai et al., 2009).

## 3 ${\dot{V}}_{A}\text{--}\dot{Q}$ RELATIONSHIPS IN THE HEALTHY LUNG: AN EVOLUTIONARY TRAIT?

Even though FRC decreases substantially between the upright and horizontal position during spontaneous breathing, pulmonary gas exchange is improved in the latter, as indicated by a reduction in the alveolar–arterial oxygen difference, and a corresponding increase in pulmonary diffusing capacity with no apparent differences between the supine and prone position (Lin et al., 2005; Rohdin et al., 2003a; Stokes et al., 1981; Terzano et al., 2009). As will be outlined below, differences between the supine and prone posture in ${\dot{V}}_{A}\text{--}\dot{Q}$ relationships are nonetheless present.

### 3.1 Distribution of ventilation during spontaneous breathing

The gradual reduction in alveolar expansion in the ventral‐to‐dorsal direction at supine FRC expectedly causes regional ventilation to increase in the same direction, as documented in several studies using a wide range of methods (Table 1). In the majority of studies, a gravitational shift in the distribution of ventilation was observed when moving to the prone posture, so that ventilation became higher ventrally. However, one study based on single‐photon emission computed tomography (SPECT) questioned these findings, as it was found that the observed posture‐dependent shift in ventilation was primarily caused by the gravitational shift in lung tissue density described above (Petersson et al., 2007). Hence, by use of transmission tomography, SPECT‐based ventilation on a ‘per alveolus’ basis was found to be unaffected (Petersson et al., 2007). When using MRI‐based methodology which has a much higher spatial resolution than SPECT, a shift in ventilation is nonetheless evident, even when lung tissue density changes are taken into account (Henderson et al., 2013). This encompasses a shift from a supine ventral‐to‐dorsal ventilation gradient to a minuscule dorsal‐to‐ventral gradient in the prone posture.

**TABLE 1:** Studies on postural shifts in ventilation and perfusion in awake healthy humans

Study	*n*	Method	Supine vertical gradient	Prone vertical gradient	Posture‐dependent gravitational shift
Ventilation					
Kaneko et al. (1966)	3	Radiospirometry (^133^Xe)	VD	DV	Yes
Rehder et al. (1978)	5	Radiospirometry (^133^Xe)	VD	DV	Yes
Orphanidou et al. (1986)	2	SPECT (^81m^Kr)	VD	VD	Yes*
Mure et al. (2001)	8	SPECT (^99m^Tc‐ DTPA)	VD	VD	No
Musch et al. (2002)	6	PET (pulmonary ^13^N_2_ elimination)	VD	DV	Yes
Petersson et al. (2007)	7	SPECT (^99m^Tc‐Technegas)	VD	VD	No
Henderson et al. (2013)	7	MRI‐SVI	VD	DV	Yes
Perfusion					
Kaneko et al. (1966)	3	Radiospirometry (^133^Xe)	VD	DV	Yes
Amis et al. (1984)	3	Radiospirometry (^85m^Kr)	None	DV	Yes
Orphanidou et al. (1986)	2	SPECT (^81m^Kr)	VD	DV	Yes
Nyrén et al. (1999)	8	SPECT (^99m^Tc‐MAA)	VD	None	Yes
Mure et al. (2001)	8	SPECT (^99m^Tc‐MAA)	VD	VD	Yes*
Jones et al. (2001)	6	Electron‐beam CT	VD	DV	Yes
Musch et al. (2002)	6	PET (^13^N_2_)	VD	DV	Yes
Petersson et al. (2007)	7	SPECT (^99m^Tc‐MAA)	VD	VD	No
Prisk et al. (2007)	6	MRI‐ASL	None	None	No
Henderson et al. (2013)	7	MRI‐ASL	VD	DV	Yes

Only studies in which assessments were done both in the supine and prone position are provided. *Reduced VD gradient in the prone compared to the supine position. ASL, arterial spin labelling; CT, computed tomography; DTPA, diethylenetriamine penta‐acetic acid; DV, dorsal‐to‐ventral; MAA, macroaggregated albumin; MRI, magnetic ressonance imaging; PET, positron emission tomograpky; SPECT, single‐photon emission computed tomography; SVI, specific ventilation imaging; VD, ventral‐to‐dorsal.

### 3.2 Distribution of pulmonary perfusion during spontaneous breathing

Posture‐dependent differences in pulmonary perfusion have been examined in spontaneously breathing supine and prone humans using several different methods (Table 1). Most studies have found that a ventral‐to‐dorsal perfusion gradient is present in the supine position, so that perfusion is higher below than above the heart. Although findings vary between studies, forces are at play that serve to prevent this from being converted to an equal dorsal‐to‐ventral gradient in the prone position.

### 3.3 ${\dot{V}}_{A}/\dot{Q}$ ratio during spontaneous breathing

**FIGURE 3:**
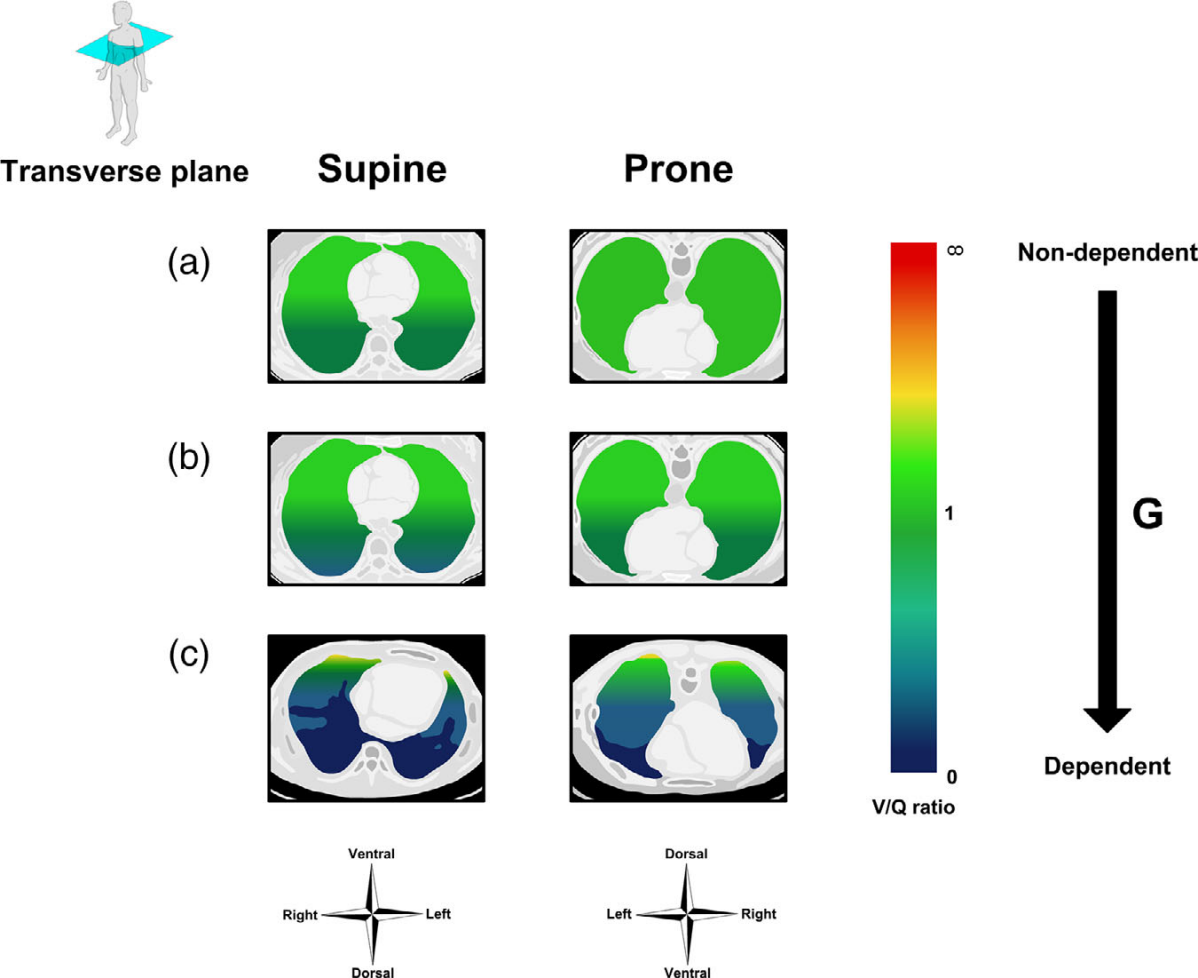
Impact of posture on the vertical distribution of the ventilation/perfusion (${\dot{V}}_{A}/\dot{Q}$) ratio in the horizontal lung. (a) The healthy lung during spontaneously breathing wakefulness. (b) The healthy lung during anaesthesia and mechanical ventilation. (c) Acute respiratory distress syndrome (ARDS) during mechanical ventilation

According to SPECT and PET‐based studies, no consistent change in the ${\dot{V}}_{A}/\dot{Q}$ ratio is observed in the vertical direction, neither in the supine or the prone lung, when the vertical tissue gradient is taken into account (Musch et al., 2002; Petersson et al., 2007). However, the high spatial resolution of MRI unveils a small albeit consistent vertical gradient, with a decline in the ${\dot{V}}_{A}/\dot{Q}$ ratio from gravitationally non‐dependent to dependent lung regions in both postures, and with slightly improved ${\dot{V}}_{A}\text{--}\dot{Q}$ matching in the prone position (Henderson et al., 2013) (Figure 3a).

### 3.4 Ventilation and perfusion in Mammalia

The postural changes in ${\dot{V}}_{A}\text{--}\dot{Q}$ relationships are evident in various Mammalia, regardless of the typical posture of the animal. Hence, postural shifts in ventilation similar to those in humans are observed in the sloth and the dog, even though the typical posture of the two is supine and prone, respectively (Hoffman & Ritman, 1985). In terms of perfusion, this has been studied in various species by the so‐called microsphere injection technique. Thus, excised lung from dog, sheep and horse demonstrate relatively uniform pulmonary perfusion distribution after microsphere injection when in their natural prone posture in vivo, while a substantial ventral‐to‐dorsal perfusion increase is evident when they are supine, regardless of whether the excised lung is prepared and studied using the same orientation as in vivo (i.e. prone) or not (Glenny et al., 1991; Hlastala et al., 1996; Walther et al., 1997). In baboons, which, like humans, are mostly upright, perfusion is likewise less uniform in the supine than in the prone posture (Glenny et al., 1999).

The greater gravity‐dependence of perfusion in the supine than in the prone posture in mammals is likely caused by a greater vascular density dorsally (Beck & Rehder, 1986; Nyrén et al., 2010). This arrangement counteracts the impact of gravity on pulmonary perfusion in the prone position, an adaptation that probably reflects that the first terrestrial mammals that appeared more than 200 million years ago were obligate quadrupeds and thus naturally prone. Given that gross lung shape and structure remain remarkably conserved across extant mammalian species, the smaller vertical ${\dot{V}}_{A}\text{--}\dot{Q}$ gradients in the prone compared to the supine posture observed in awake, spontaneously breathing humans likely reflect that the mammalian lung is phylogenetically set to function optimally in the prone posture.

## 4 PRONING, GENERAL ANAESTHESIA, AND MECHANICAL VENTILATION: A GREAT MATCH?

In the clinical setting, proning is mostly instigated in anaesthetized and mechanically ventilated patients. Mechanical ventilation imposes a positive airway pressure, which may affect the ${\dot{V}}_{A}\text{--}\dot{Q}$ distribution per se, while sedation causes the diaphragm to deviate cranially, presumably due to a loss of respiratory muscle tone (Froese & Bryan, 1974; Krayer et al., 1989; Rehder et al., 1977). The latter reduces FRC by approximately 15–20% compared to awake supine breathing in the same position (Froese & Bryan, 1974; Krayer et al., 1989; Rehder et al., 1977). Consequently, posture affects pulmonary gas exchange somewhat differently than in the awake and spontaneously breathing state.

### 4.1 Effects of anaesthesia on ventilation

In anaesthetised healthy individuals placed in the supine position, ventilation exhibits a largely similar distribution to spontaneously breathing wakefulness in the same position (Table 2). However, a successive decrease in ventilation occurs in the dorsal regions during prolonged anaesthesia due to a degree of airway collapse (Nyrén et al., 2010; Tokics et al., 1996). Accordingly, attempts to open collapsed alveoli by applying a sustained positive end‐expiratory pressure (PEEP) reestablishes the vertical ventilation gradient (Petersson et al., 2010). During mechanical ventilation in the prone position, ventilation is shifted dorsally (Table 2), and when a PEEP is applied, ventilation is shifted in the ventral direction (Petersson et al., 2010).

**TABLE 2:** Studies on postural shifts in ventilation and perfusion in anaesthetized healthy humans

Study	*n*	Method	Supine vertical gradient	Prone vertical gradient	Posture‐dependent gravitational shift
Ventilation					
Rehder et al. (1978)	5	Radiospirometry (^133^Xe)	VD	DV	Yes
Petersson et al. (2010)	6	SPECT (^99m^Tc‐Technegas)	DV	VD	Yes
Nyrén et al. (2010)	7	SPECT (^99m^Tc‐Technegas)	VD	VD	No
Perfusion					
Petersson et al. (2010)	6	SPECT (^99m^Tc‐MAA)	VD	VD	Yes*
Nyrén et al. (2010)	7	SPECT (^113m^In‐Technegas)	VD	None	Yes

Only studies in which assessments were done both in the supine and prone position are provided. *Reduced VD gradient in the prone compared to the supine position. DV: dorsal‐to‐ventral; MAA: macroaggregated albumin; SPECT: single‐photon emission computed tomography; VD: ventral‐to‐dorsal.

### 4.2 Effects of anaesthesia on perfusion

During anaesthesia in the supine position, a perfusion gradient from the gravitationally non‐dependent ventral to dependent dorsal lung is observed. When assuming the prone postion, perfusion either remains highest in the now non‐dependent dorsal lung regions or the vertical perfusion gradient is abolished altogether (Table 2). Nonetheless, when a PEEP of 10 cm H_2_O is applied, perfusion is redistributed to the gravitationally dependent part of the lung in both the supine and prone posture, conceivably due to compression of blood vessels in non‐dependent lung regions (Petersson et al., 2010).

### 4.3 Effects of anaesthesia and PEEP on the ${\dot{V}}_{A}/\dot{Q}$ ratio

A vertical ${\dot{V}}_{A}/\dot{Q}$ ratio gradient is observed during anaesthesia and mechanical ventilation in both the supine and the prone position, which decreases in the ventral‐to‐dorsal direction in the former and in the dorsal‐to‐ventral direction in latter position (Nyrén et al., 2010; Petersson et al., 2010). However, the gradient is lowest in the prone position (Figure 3b).

In the supine posture, ${\dot{V}}_{A}\text{--}\dot{Q}$ relationships are not affected when a PEEP of 10 cmH_2_O is applied, since ventilation and perfusion are redistributed similarly towards the gravitationally dependent dorsal lung regions (Petersson et al., 2010). However, in the prone position, the application of similar PEEP levels renders ${\dot{V}}_{A}\text{--}\dot{Q}$ relationships suboptimal by redistributing ventilation towards ventral lung regions to a much greater degree than perfusion (Petersson et al., 2010).

In summary, ${\dot{V}}_{A}\text{--}\dot{Q}$ matching is improved upon a postural change from supine to prone in healthy anaesthetised individuals, due to a shift of both ventilation and perfusion towards the gravitationally non‐dependent dorsal lung areas. This may indeed improve pulmonary gas exchange, as some, but not all, studies of anaesthetised and mechanically ventilated healthy individuals show notable $P_{aO_{2}}$ increments within 30 min of proning (Pelosi et al., 1995, 1996; Petersson et al., 2010; Soro et al., 2007; Stone & Khambatta, 1978).

## 5 DESTRESSING THE DISTRESSED ARDS LUNG BY PRONING: A TURN FOR THE BETTER

Several studies on mechanically ventilated patients with ARDS have provided evidence of improved pulmonary gas exchange in response to proning as evaluated by an increased $P_{aO_{2}}$/$F_{IO_{2}}$ ratio, possibly persisting even after resupination (Mure et al., 1997; Pappert et al., 1994; Gattinoni et al., 2001; Guérin et al., 1999, 2004, 2013; Lee et al., 2002; Mancebo et al., 2006; Taccone et al., 2009). However, although approximately 70% of ARDS patients exhibit a $P_{aO_{2}}$ increase of ≥10 mmHg within 30 min after proning, improvements in blood gases did not readily explain the improved survival observed in the PROSEVA trial (Albert et al., 2014; Guérin et al., 2013).

### 5.1 Pathogenesis and pathophysiology of ARDS

The histopathological hallmark of ARDS is diffuse alveolar damage with protein‐rich alveolar oedema and sequestration of immune cells in the interstitial and alveolar spaces (Matthay et al., 2012). This renders the lung less compliant with a four to five time increase in its mass, so that the superimposed pressure increases the pleural pressure gradient substantially with consequent severe compression atelectasis in gravitationally dependent lung regions (Crotti et al., 2001; Gattinoni et al., 1988, 1994, 2006; Pelosi et al., 1994). Apart from the increased lung mass, an additional contributor is probably also a notable increase in cardiac mass, which may double, and in some cases also abdominal distension (Albert & Hubmayr, 2000; Malbouisson et al., 2000; Mure et al., 1998).

Imaging studies of the pulmonary ${\dot{V}}_{A}\text{--}\dot{Q}$ relationships in ARDS are scarce, but the physiological consequences of the severely atelectatic and oedematous lung regions have been elucidated by the multiple inert gas elimination technique (Dantzker et al., 1979; Ralph et al., 1985). In these studies, the main mechanism of impaired pulmonary gas exchange was found to be intrapulmonary shunting, which often exceeded 30% of cardiac output.

### 5.2 Proning in ARDS

When patients with ARDS are moved from the supine to the prone position, atelectatic areas shift from dorsal to ventral regions, so that the dorsal lung regions receive (near‐)normal aeration (Gattinoni et al., 1991; Guérin et al., 1999), while the FRC also increases (Aguerre‐Bermeo et al., 2018), and the shunt fraction typically decreases by approximately 10 percentage points (Lee et al., 2002; Pappert et al., 1994). However, the associated changes in ${\dot{V}}_{A}\text{--}\dot{Q}$ relationships have not been examined in humans.

In animal studies of experimental ARDS triggered either by intrapulmonary oleic acid injection or by surfactant depletion, a reduction in intrapulmonary shunt similar to that of ARDS patients has been observed in the prone compared to the supine posture (Lamm et al., 1994; Richter et al., 2005; Wiener et al., 1990). The atelectatic regions in gravitationally dependent lung regions are the principal cause of intrapulmonary shunting in both postures (Lamm et al., 1994; Richter et al., 2005). Hence, in the supine posture, severe ${\dot{V}}_{A}\text{--}\dot{Q}$ mismatching is evident with a steep ventral‐to‐dorsal reduction in ventilation, including entirely absent ventilation in the most dorsal atelectatic areas, and with a concomitant ventral‐to‐dorsal increase in perfusion (Lamm et al., 1994; Richard et al., 2008; Richter et al., 2005; Scaramuzzo et al., 2020; Wiener et al., 1990). In the prone position, ventilation and to a lesser extent perfusion both become more uniformly distributed with less steep vertical gradients, and although collapse occurs in the now gravitationally dependent ventral lung regions, it is much reduced (Lamm et al., 1994; Richard et al., 2008; Richter et al., 2005; Scaramuzzo et al., 2020; Wiener et al., 1990) (Figure 3c).

However, proning does not appear to be sufficient to reduce intrapulmonary shunting per se. According to PET‐based studies on piglets with oleic acid‐induced lung injury, PEEP was found to be a necessary prerequisite to achieve this (Richard et al., 2008). Indeed, the application of PEEP is the main therapeutic procedure for reducing atelectasis in ARDS, mainly by maintaining alveolar patency at end‐expiration (Crotti et al., 2001; Gattinoni et al., 2006; Ralph et al., 1985). However, the ideal PEEP titration strategy, i.e., achieving optimal alveolar ventilation while preventing hyperinflation, is still not clear.

Together, the available animal studies indicate that the postural improvements in pulmonary ${\dot{V}}_{A}\text{--}\dot{Q}$ relationships between the supine and prone position in ARDS are mostly driven by a redistribution of ventilation to the dorsal region of the lungs and less atelectasis. Furthermore, the reduction in the pleural pressure gradient imposed by proning will reduce the PEEP required to maintain alveolar patency. Accordingly, proning is mainly thought to offer a survival benefit in ARDS by rendering the intrapulmonary distribution of tidal volume more homogeneous, thus preventing alveolar (over‐)distension of non‐atelectatic lung tissue, and reducing the risk of ventilator‐induced lung injury (Albert et al., 2014; Hepokoski et al., 2018).

## 6 COVID‐19‐ASSOCIATED RESPIRATORY FAILURE

Both ARDS and CARDS are associated with interstitial and alveolar accumulation of immune cells (Matthay et al., 2018; Ronit et al., 2021), but a distinct feature prominent in CARDS is pulmonary micro‐ and macrovascular disease with in situ thrombosis and/or thromboemboli (Ackermann et al., 2020). This sets in from the early stages of disease,indicated by chest CT findings typical of pulmonary vascular disease, such as ground glass opacities, septal thickening and linear opacities, which is currently thought to be a main contributor to the conspicuous hypoxaemic respiratory failure of CARDS (Rubin et al., 2020; Simonson et al., 2021; Wu et al., 2020).

The pulmonary predilection for thrombosis and thromboemboli appears to be related to the presence of severe pulmonary vasculitis, most likely because of viral invasion of the endothelium and the precipitation of immune complexes in the vasculature (Ackermann et al., 2020; Roncati et al., 2020). Accordingly, a relatively large proportion of CARDS patients have been reported to present with severe hypoxaemia despite near‐normal pulmonary compliance (and lung weight), a combination that is otherwise rare in ARDS (Gattinoni et al., 2020; Rello et al., 2020). Nonetheless, with the progression of disease, many patients with moderate to severe CARDS eventually exhibit a phenotype that is clinically indistinguishable from non‐COVID‐19 ARDS (Gattinoni et al., 2020; Rello et al., 2020).

### 6.1 Proning in CARDS

The initial clinical experience with proning in CARDS showed a marked increase in the $P_{aO_{2}}$/$F_{IO_{2}}$ ratio of ∼60 mmHg (Carsetti et al., 2020; Pan et al., 2020; Perier et al., 2020), and findings from a recent observational study furthermore suggest that proning is independently associated with improved 28‐day survival in this setting (Ferreira et al., 2021). Studies based on electrical impedance tomography in CARDS have reported that, similar to patients with ARDS, proning shifts ventilation dorsally, and because perfusion remains predominantly in the dorsal lung regions, ${\dot{V}}_{A}\text{--}\dot{Q}$ matching is improved (Perier et al., 2020; Zarantonello et al., 2020).

When considering the effects of proning on healthy anaesthetised humans, the postural shift in the ${\dot{V}}_{A}\text{--}\dot{Q}$ distribution may also exert clinical benefits in the hypoxaemic CARDS patient with normal lung weight and compliance. Indeed, to prevent ICU transfers in overloaded health systems, proning is now also used even in non‐intubated COVID‐19 patients with hypoxaemic respiratory failure, in which it is generally well tolerated and may lead to substantial increases in $P_{aO_{2}}$ (Damarla et al., 2020; Elharrar et al., 2020).

## 7 CONCLUSION AND FUTURE DIRECTIONS

The human lung, as with other mammalian lungs, is structurally optimised to function in the prone posture, both in terms of both gross lung shape and vascular architecture. Consequently, ventilation and perfusion are better matched in the prone than in the supine posture despite an identical gravitational vector. Thus, in the supine position, gravity and lung structure cause regional ventilation and perfusion to diverge, and this posture is thus suboptimal in disease states with pathological ${\dot{V}}_{A}\text{--}\dot{Q}$ mismatching, such as ‘classic’ ARDS and CARDS. The improvement in arterial oxygenation observed upon proning in these conditions likely reflects that the lung assumes its phylogenetically ‘natural’ posture.

While the impact of proning on mortality is well‐established in classical ARDS, mainly due to lung protective effects involving a reduced risk of ventilator‐induced lung injury, the data regarding CARDS are still limited, including whether the associated improvement in oxygenation offers any survival benefit per se. Proning is nevertheless an attractive therapeutic intervention for providing lung protective ventilation and alleviating life‐threatening hypoxaemia rapidly, effectively, and safely. A better understanding of the effects of proning on ${\dot{V}}_{A}\text{--}\dot{Q}$ relationships as well on clinically relevant endpoints, such as the need for intubation, mortality and length of ICU or hospital stay, is, however, necessary and should be explored in prospective clinical studies.

## COMPETING INTERESTS

None of the authors have any conflict of interest to disclose.

## AUTHOR CONTRIBUTIONS

All authors were involved in the conception and/or design of the study, acquisition and/or analysis, drafting the work and/or revising it critically for important intellectual content. All authors have read and approved the final version of this manuscript and agree to be accountable for all aspects of the work in ensuring that questions related to the accuracy or integrity of any part of the work are appropriately investigated and resolved. All persons designated as authors qualify for authorship, and all those who qualify for authorship are listed.
